# Modulation of Gut Microbiota in Korean Navy Trainees following a Healthy Lifestyle Change

**DOI:** 10.3390/microorganisms8091265

**Published:** 2020-08-20

**Authors:** YeonGyun Jung, Setu Bazie Tagele, HyunWoo Son, Jerald Conrad Ibal, Dorsaf Kerfahi, Hyunju Yun, Bora Lee, Clara Yongjoo Park, Eun Soo Kim, Sang-Jun Kim, Jae-Ho Shin

**Affiliations:** 1 School of Applied Biosciences, Kyungpook National University, Daegu 41566, Korea; jyg1076@knu.ac.kr (Y.J.); setubazie@gmail.com (S.B.T.); thsgusdn2@knu.ac.kr (H.S.); jerald.ibal@gmail.com (J.C.I.); 2 Department of Applied Plant Sciences, University of Gondar, Gondar 196, Ethiopia; 3 Department of Biological Sciences, Keimyung University, Daegu 42601, Korea; kerfahi.dorsaf@gmail.com; 4 Department of Food and Nutrition, Chonnam National University, Gwangju 61186, Korea; virt0323@naver.com (H.Y.); lbr1074@naver.com (B.L.); parkcy@jnu.ac.kr (C.Y.P.); 5 Department of Internal Medicine, School of Medicine, Kyungpook National University, Daegu 41944, Korea; dandy813@hanmail.net; 6 Department of Natural Sciences, Republic of Korea Naval Academy, Changwon 51702, Korea; sjkim1963@navy.mil.kr

**Keywords:** gut microbiome, lifestyle, smoking cessation, alpha-diversity, beta-diversity, *Bifidobacterium*

## Abstract

Environmental factors can influence the composition of gut microbiota, but understanding the combined effect of lifestyle factors on adult gut microbiota is limited. Here, we investigated whether changes in the modifiable lifestyle factors, such as cigarette smoking, alcohol consumption, sleep duration, physical exercise, and body mass index affected the gut microbiota of Korean navy trainees. The navy trainees were instructed to stop smoking and alcohol consumption and follow a sleep schedule and physical exercise regime for eight weeks. For comparison, healthy Korean civilians, who had no significant change in lifestyles for eight weeks were included in this study. A total of 208 fecal samples were collected from navy trainees (*n* = 66) and civilians (*n* = 38) at baseline and week eight. Gut flora was assessed by sequencing the highly variable region of the 16S rRNA gene. The α-and β -diversity of gut flora of both the test and control groups were not significantly changed after eight weeks. However, there was a significant difference among individuals. Smoking had a significant impact in altering α-diversity. Our study showed that a healthy lifestyle, particularly cessation of smoking, even in short periods, can affect the gut microbiome by enhancing the abundance of beneficial taxa and reducing that of harmful taxa.

## 1. Introduction

The human gut microbiota is a reservoir of microorganisms that plays a crucial role to human health [1] through their involvement in metabolic interactions (e.g., food decomposition and nutrient intake) [2,3], drug metabolism [4,5], energy production and storage [6], and protection against pathogens [7]. The gut microbiome provides signals that influence the development of the host immune system and stimulate the maturation of immune cells [8,9]. More importantly, the gut microbiota is not only associated with human well-being but also with human disease conditions, including metabolic diseases, growth disorders, mental illness (e.g., autism), and obesity [10,11,12,13,14]. Consequently, gut microbes affect human physiology both directly and indirectly [7]. Abrupt changes to the delicate balance of the microbial assemblage can result in unexpected consequences [15]. For this reason, it is necessary to understand the structure of microbial communities and the factors that modulate this community structure.

Microbial establishment in the human intestine begins at birth [1,6]. Subsequently, the intestinal microflora continues to develop through successive microbial communities, until the microbial climax community colonizes the intestine [4,5]. Moreover, because of co-evolutionary interactions, microbes undergo further functional modifications [16]. Between one and two years after birth, the intestinal microbial community becomes more complex and stabilizes to an adult-like structure [17,18,19]. Early events such as birth mode, the presence of siblings, type of infant feeding, and the use of antibiotics at birth can affect the formation of gut microbiota because of the relatively low diversity of intestinal microbial communities in infancy [20].

Understanding the stability of human gut microbiota plays a key role in determining the dynamics of gut microbiota and advancing personalized therapies [21]. Intestinal microbial communities established in most adults remain stable [21]. However, the mechanism of microbiota stability in the gut remains unknown [5]. A previous study demonstrated that samples obtained over time from the same individual were more similar to each other than samples from other individuals and that each individual had a relatively distinct and stable community [14]. Similarly, the gut microbiota is reported to be temporarily stable based on self-restoration of microbiota after disturbance [22,23].

However, several factors including lifestyle, diet, stress, and probiotics have been implicated in altering the gut microbiota [24,25,26]. Beta diversity was significantly impacted by cigarette smoking [27], and physical activity was also reported to potentially alter the relative abundance of the Firmicutes, Bacteroidetes, and Proteobacteria phyla [28]. In addition, drinking, sleeping, and body weight are also known to affect the intestinal microbiota in adults [29,30,31]. However, the role of combined modifiable lifestyle factors on gut microbiota of adults remains unexplained. Investigating the impact of changes in lifestyle factors on gut microbiota could contribute to effectively preventing health risks associated with dysbiosis. The gut microbiota is a key determinant of human health [32]. Hence, there is a need to investigate the relationship between combined lifestyle interventions and gut microbiota. In this study, we investigated the relationship between modifiable lifestyle factors of South Korean naval trainees and the community structure and diversity of their intestinal microbial communities. We performed next-generation sequencing of 16S rRNA genes to analyze the gut flora of naval trainees during the trainee period when the trainees all experienced similar environmental conditions in the naval officer candidate school (OCS).

## 2. Materials and Methods

### 2.1. Recruitment of Subjects and Sampling

The present study was approved by the Institutional Review Board of Kyungpook National University (KNU 2017-84) (24-08-2017), and the Armed Forces Medical Research Ethics Review Committee (AFMC-17-IRB-092) (17-11-2017), Republic of Korea. All subjects gave written informed consent in accordance with the Declaration of Helsinki. 

Subjects were recruited on the first day of training admission to the OCS. The trainees lived in the same environment for eight weeks, ate the same food at regular intervals, and participated in similar training and sleeping regimes. They were also not allowed to smoke or drink for eight weeks. As a control, healthy people living in Korea at the same sampling points as the OCS were also recruited as subjects. The civilian group did not change their lifestyle habits for the eight weeks.

Fecal samples were collected from 66 trainees of the naval OCS on the first day of enlistment (week zero) and eight weeks after admission to the naval center (week eight). As controls, fecal samples were also collected from 38 healthy people living in Korea at the same sampling points as the OCS. All samples were collected by participants using Transwab tubes (Sigma, Dorset, UK) and sent to the laboratory, where they were stored at −80 °C until DNA extraction.

### 2.2. Data Collection

Participants were asked to complete a self-administered questionnaire to collect demographic, lifestyle, and physical activity data at week zero (Table S1). Dietary consumption was assessed by a food frequency questionnaire (FFQ) used in the 2017 Korea National Health and Nutrition Examination Survey conducted by the Korea Centers for Disease Control and Prevention. The FFQ was completed by participants. Reported intakes below 500 kcal/d or > 5000 kcal/d for civilians and > 6000 kcal/d for navy trainees were determined inaccurate and excluded from further analyses. The OCS provided naval trainees’ menus, which were analyzed for nutrient content using the computer-aided nutritional analysis program (CAN Pro 5.0, Korea Nutrition Society, Seoul, Korea). The daily nutrient intake for foods consumed by trainees that were not available in CAN Pro 5.0 were calculated by referencing the Korean Food Composition Database, Version 9.1. Meals were served buffet style, thus the analyzed nutrients were based on the ideal diet intake for trainees. Menus from five days immediately prior to each naval trainees’ fecal collection date were used to estimate mean daily nutrient intake for the eight weeks of the study for the naval trainees.

Subjects were classified into subgroups for each lifestyle based on the content of the questionnaire obtained from the subjects at week zero (Table 1). The method described by Ryan et al. [33] was used to classify smoking, alcohol consumption, physical exercise, and body mass index. Sleep time was classified according to the National Sleep Foundation [34].

### 2.3. DNA Extraction, PCR Amplification, and Sequencing

Genomic DNA was extracted from approximately 500 μL (wet weight) of each sample using QIAamp PowerFecal DNA Isolation kits (Qiagen, Hilden, Germany). Extracted DNA was assessed for quality by electrophoresis and was quantified using a Qubit 2.0 Fluorometer (Life Technologies, Carlsbad, CA, USA). DNA isolated from each sample was amplified using the universal primers, 515 F (5′-barcode-GTGCCAGCMGCCGCGGTAA-3′) and 907 R (5′-barcode-CCGYCAATTCMTTTRAGTTT-3′), targeting the V4-V5 regions of prokaryotic 16S rRNA genes. The barcode was an eight-base sequence unique to each sample. PCR experiments were performed under the following conditions: 95 °C for 5 min, 30 cycles of 95 °C for 30 s, 57 °C for 30 s, 72 °C for 30 s, and then 72 °C for 5 min and held at 4 °C. PCR was performed in duplicate in 24 μL reaction volumes, consisting of 20 μL Emerald AMP GT PCR 1× Master Mix (Takara Bio, Shiga, Japan), 0.5 μL (10 μM) of each barcoded PCR primer pair, and 3 μL of DNA template (10–50 ng DNA). PCR products were purified using an AMPure XP bead purification kit (Beckman Coulter, Brea, CA, USA) and pooled in equal concentrations. An Agilent 2100 Bioanalyzer (Agilent Technologies, Santa Clara, CA, USA) was used to confirm the correct concentration needed for sequencing. Each amplified region was sequenced on an Illumina MiSeq sequencing platform (Illumina, San Diego, CA, USA) using a MiSeq Reagent Kit v3 (Illumina, Inc., San Diego, CA, USA), according to the manufacturer’s protocols.

### 2.4. Bioinformatic Analysis

16S rRNA amplicon primers were removed from sequencing reads using cutadapt version 2.8 [35]. The primer-trimmed files were imported into Quantitative Insights Into Microbial Ecology 2 (QIIME2) v. 2020.2 software [36] in Casava 1.8 single-end demultiplexed format for further processing using different algorithms (implemented in QIIME2). Quality-filtered reads were then input into the QIIME2 plugin Deblur [37] to produce amplicon sequence variants (ASV). A trim length of 200 base pairs was used, and the minimum number of reads required to pass filtering was set to 1; ASVs that were found in an abundance of < 0.1% of the mean sample depth were then removed from analysis. After filtering a total of 12,509 ASVs were recovered. The sequences were filtered to remove non-bacterial, mitochondrial, and chloroplast sequences. Representative sequences were then assigned taxonomy using a custom trained V4–V5 16S rRNA naive Bayesian QIIME2 classifier [38] trained on the 99% Silva V132 database [39]. The mafft, mask and FastTree protocols were then used to generate rooted and unrooted phylogenetic trees of aligned representative sequences for use in diversity analysis. The final feature table was rarefied to 3124 reads per sample. Sample diversity metrics were generated for α-diversity and β-diversity.

### 2.5. Statistical Analysis

The D’Agostino-Pearson Omnibus test was used to determine the distribution of data in RStudio 1.0.153 (https://www.rstudio.com/). Statistical analysis was performed by Kruskal–Wallis tests for multiple comparisons. Wilcoxon matched-pairs signed-rank was assessed to compare the differences in α-diversity across subgroups and over time. Analyses were performed using Prism 8 software (GraphPad Software, San Diego, CA, USA). A *p*-value of <0.05 was considered significant; data were tested to determine whether diversity indices were significantly different between samples collected at different time points. Statistical analyses for β-diversity were completed by calculating Bray–Curtis distance using QIIME2. A permutational multivariate analysis of variance (PERMANOVA) was run to complete pairwise comparisons of samples for each subgroups. The heatmap of the top 30 genera of each subject were performed in R 3.6.3 using the packages phyloseq [40], qiime2R [41], and vegan (version 2.5-6) [42]. The edgeR package [43] was used to evaluate univariate differential abundance of operational taxonomic units (OTUs). OTUs having a false discovery rate (FDR) < 0.05 were considered as differentially abundant. Contrast analyses were performed between T0 and T8 to generate statistical differences from the OTU sequences selected by the edgeR package version 3.16. Figures were created in R using ggplot2 [44], ggpubr [45], and VennDiagram [46].

## 3. Results

### 3.1. Characterstics of the Participants and Data Summary of Amplicon Sequencing

To determine whether a change in lifestyle influenced gut microbial community structure and diversity, 16S rRNA-based metagenomic profiling of fecal samples collected from civilians and navy trainees was performed. The samples were collected on the first day of admission to the OCS (baseline, T0) and eight weeks after admission (T8). The trainees were instructed to stop smoking and drinking alcohol during the eight week training period. In addition, the trainees were expected to maintain the sleep schedule and time of physical exercise. Physical exercise was performed every day for eight weeks through basic military training, and cardio (mean = 255.15, Interquartile range (IQR) = 132.50–245.00) and weight training (mean = 39.78, IQR = 10.00–24.50) were performed together. The navy trainees were exposed to the same lifestyle at the naval OCS for eight weeks. The civilian group had no significant lifestyle change during the eight week study (Table 2).

The intestinal microbial communities of the fecal samples of navy trainees and civilians collected at T0 and T8 were analyzed. A total of 208 fecal samples was collected from the 66 naval trainees and 38 Korean civilians. We detected 1635 features from 3,210,100 high-quality sequence reads. The average number of reads per sample was 15,433 (ranged from 3124 to 26,765). For each feature, there was a mean frequency of 1963 observations (ranging from 15 to 203,394). For downstream analysis, the frequency tables were rarified at an even sampling depth of 3124 reads per sample. At this level, 649,792 of the original sequences (20.24%) and all of the 208 samples were retained in the data set.

### 3.2. Intestinal Microbial Communities after Eight Weeks of Lifestyle Intervention

The effect of significant lifestyle changes on gut flora of navy trainees was investigated after eight weeks of intervention in comparison with civilians, who had no significant lifestyle changes for eight weeks. The relative bacterial abundance after eight weeks in the two groups is presented by the heat map at genus level (Figure S1a). Alpha diversity indices including the Shannon index, observed OTUs, Faith’s phylogenetic diversity (PD), and evenness index were calculated at T0 and after T8 (Figure S1b). The diversity indices did not significantly differ between T0 and T8 in either the navy trainee group or the civilian group (Figure S1b). We also performed Bray–Curtis principal-coordinate analysis (PCoA) ordination to determine the β-diversity of the intestinal microbial communities of the navy trainee and civilian groups at T0 and T8. The PCoA plot showed that intestinal microbial communities at T0 and T8 were not separate to each other in each group (Figure S1c). However, there were significant differences among individuals (Table 3).

**Figure 1:**
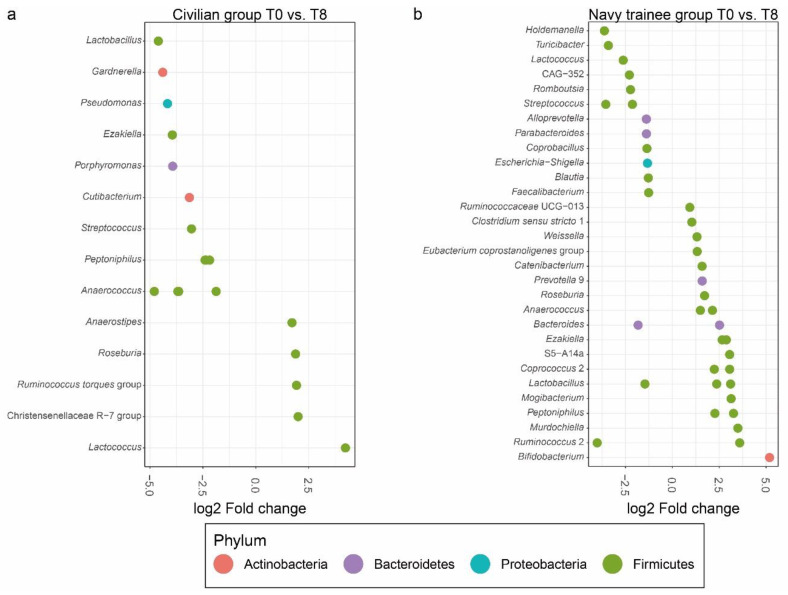
Differential abundance analysis of amplicon sequencing data of the samples from (**a**) civilian and (**b**) navy trainee groups after eight weeks using the edgeR package. Operational taxonomic units (OTUs) which significantly distinguished either navy trainee or civilian groups at week zero and week eight are presented. Only OTUs with a false discovery rate (FDR) value < 0.05 were considered as being differentially abundant. Each point represents an OTU belonging to the respected genera. Points in different colors represent different phylum.

To observe microbial changes at lower taxa levels after eight weeks in each group, edgeR was used for contrast analysis. Among the 1635 tested features, 20 and 39 taxa were found to be significantly different between the civilian and navy trainee groups, respectively (Figure 1 and Table S2). In the civilian group, the majority (80%) of OTUs that had undergone changes over eight weeks belonged to the Firmicutes phylum, followed by Actinobacteria (10%), Proteobacteria (5%), and Bacteroidetes (5%) (Figure 1a). In this group, OTUs belonging to the *Anaerococcus* genus were the most decreased (LogFC = −4.80, *p* = 8.85 × 10^−10^), whereas the *Lactococcus* genus had the most increased OTUs (LogFC = 4.24, *p* = 5.56 × 10^−12^) at T8 in comparison with OTUs at T0 (Figure 1a). In the navy trainee group, the majority (82%) of OTUs that had undergone changes over eight weeks belonged to the Firmicutes phylum, followed by Bacteroidetes (13%), Proteobacteria (3%), and Actinobacteria (3%) (Figure 1b). OTUs belonging to the *Ruminococcus* 2 (LogFC = −4.01, *p* = 1.02 × 10^−12^), *Holdemanella* (LogFC = −3.62, *p* = 1.76 × 10^−11^), *Streptococcus* (LogFC = −3.55, *p* = 1.46 × 10^−9^ / LogFC = −2.13, *p* = 2.80 × 10^−8^), and *Turicibacter* genera (LogFC = −3.41, *p* = 7.91 × 10^−13^) were significantly reduced, whereas those of *Bifidobacterium* (LogFC = 5.18, *p* = 7.38 × 10^−27^) and *Murdochiella* genera (LogFC = 3.49, *p* = 1.28 × 10^−14^) were found to be significantly increased at T8 compared with OTUs at T0 (Figure 1b).

### 3.3. Effects of Modifiable Lifestyles Factors on Diversity of Intestinal Microbial Communities

**Figure 2:**
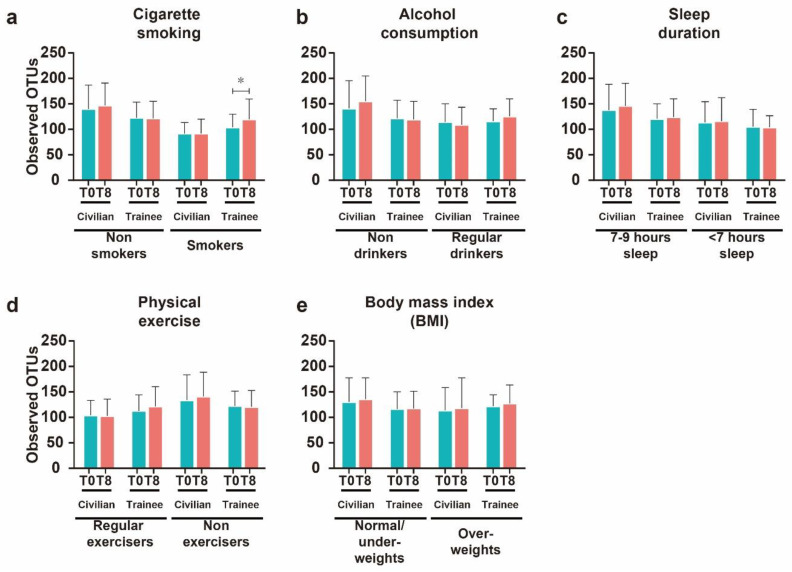
Number of observed OTUs detected at week zero (T0) and week eight (T8) in civilian and navy trainee groups. Number of OTUs at T0 and T8 in different categories: cigarette smoking (**a**), alcohol consumption (**b**), sleep duration (**c**), physical exercise (**d**), and body mass index (BMI) (**e**). Values are expressed as means ± SEM. Asterisk (*) indicates statistically significant (*p* < 0.05) differences between groups based on Wilcoxon matched-pairs signed-rank test. At T8, the navy trainees had stopped smoking and drinking alcohol, and had been instructed to do physical exercise for 5 h per day and obey the 8 h sleep schedule.

To determine if the lifestyle changes over eight weeks for the navy trainees modulated the intestinal microbial diversity, we compared the diversity indices of T0 and T8 of each lifestyle factor (Figure 2). The results showed that quitting smoking resulted a significant (*p* < 0.05) increase in the number of observed OTUs after eight weeks (Figure 2a). Smokers in the navy trainee group at T0 had low OTUs (mean = 103.90, IQR = 95.18–118.20), which were significantly higher (*p* = 0.031) (mean = 119.60, IQR = 105.30–132.30) at T8. In contrast, there was no significant difference in the navy trainee nonsmoking group in OTUs (*p* = 0.222) between T0 (mean = 122.60, IQR = 104.70–136.90) and T8 (mean = 121.30, IQR = 94.50–143.30). In civilians, regardless of smoking history, there was no significant change (*p* = 0.207) in OTUs between T0 and T8. The number of OTUs in smokers in the civilian group at T0 (mean = 91.55, IQR = 67.50–108.30) and T8 (mean = 91.75, IQR = 69.10–104.60) was not significantly different. This is because of the fact that civilian group unlike navy trainees were able to continue smoking cigarettes. As expected, the nonsmoking civilian group displayed no significant difference in OTUs (*p* = 0.242) between T0 (mean = 139.80, IQR = 104.30–177.10) and T8 (mean = 147.00, IQR = 113.70–164.60). Similarly, the remaining lifestyle factors had no impact in modulating the OTUs of civilians. In addition, the change in the other lifestyle factors after eight weeks in the navy trainees did not result in a significant change in the number of OTUs (Figure 2b–e). Navy trainees who stopped drinking alcohol for eight weeks displayed a slight increment, though this was non-significant, of OTUs at T8 (mean = 125.60, IQR = 96.60–136.90) compared with that at T0 (mean = 115.70, IQR = 103.60–122.80) (Figure 2b).

Furthermore, similar to the OTU results, the navy trainee smokers who had stopped for eight weeks had a significantly higher (5.01 ± 0.58; *p* < 0.05) Shannon index compared with that at T0 (4.81 ± 0.46) (Figure S2). However, there was no difference between T0 and T8 of navy smokers in Faith’s PD and evenness index (Figure S2). Similarly, civilians who kept smoking for eight weeks showed no significant difference in Shannon index at T8 compared with that at T0 (Figure S2). These results signify that quitting smoking was the most important factor in modulating the intestinal microbial community diversity within a short period of time. However, significant change of the remaining lifestyle factors in the eight week period (Table 2) did not significantly alter the Shannon index, Faith’s PD, or evenness index of navy trainees (Figure S2). In addition, in the civilian group, who did not have a significant (*p* > 0.2) lifestyle changes (Table 2), there was no difference in Faith’s PD or evenness index between T0 and T8 (Figure S2). Nevertheless, those in the civilian group who kept drinking alcohol for eight weeks, had a significantly (*p* < 0.05) lower Shannon index at T8 (4.59 ± 0.86) compared with that at T0 (4.77 ± 0.83) (Figure S2a). These results suggest that with the exception of stopping smoking, a significant change in healthy lifestyle factors such as physical exercise, sufficient sleep, and reducing alcohol consumption over a short period of time had no impact in modulating the intestinal microbial diversity.

In the case of beta-diversity, PERMANOVA analysis was performed using distance matrices to determine if the change in lifestyles for eight weeks significantly contributed to the difference in gut microbiota of navy trainees and civilians (Table S3). The results showed that the lifestyle changes over the eight weeks did not significantly (PERMANOVA: *p* > 0.05) alter the beta-diversity in either navy trainees or civilians.

### 3.4. Cigarette Smoking Altered the Abundance of Individual Bacterial Taxa

**Figure 3:**
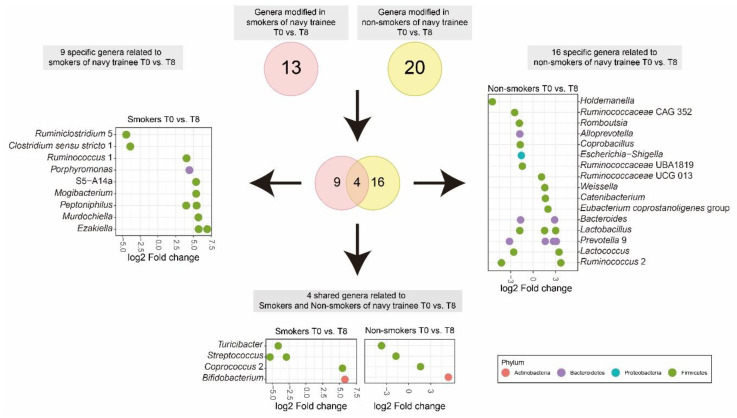
Venn diagram and Log2 fold change in the relative abundance of specific genera in smokers and non-smokers of the navy trainee group. Note: smokers had stopped smoking for eight weeks.

Among the lifestyle factors, smoking had the most significant impact in altering alpha diversity. Hence, to further investigate the impact of stopping smoking on specific bacterial taxa, we compared the log_2_ fold change of the relative abundance of individual taxa in non-smokers and smokers of navy trainees (Figure 3 and Table S4). The abundance of 13 genera (16 OTUs) was significantly (*p* < 0.001) changed in the gut of navy trainees who had previously smoked cigarettes but then quit for eight weeks. Nine of the 13 genera were significantly more abundant, whereas the abundance of four other genera was significantly reduced. Genera that were more abundant at T8 compared with abundance at T0 were: *Ruminococcus* 1 (LogFC = 4.03, *p* = 1.12 × 10^−4^), *Porphyromonas* (LogFC = 4.44, *p* = 4.63 × 10^−5^), S5-A14 (LogFC = 5.37, *p* = 5.41 × 10^−6^), *Mogibacterium* (LogFC = 5.39, *p* = 5.67 × 10^−6^), *Peptoniphilus* (LogFC = 5.45, *p* = 6.53 × 10^−6^), *Murdochiella* (LogFC = 5.70, *p* = 4.38 × 10^−6^), and *Ezakiella* (LogFC = 6.94, *p* = 6.15 × 10^−7^ / LogFC = 5.73, *p* = 2.22 × 10^−6^). In contrast, two taxa, namely *Ruminiclostridium* 5 (LogFC = −4.45, *p* = 4.22 × 10^−5^) and *Clostridium sensu stricto* 1 (LogFC = −3.88, *p* = 1.72 × 10^−3^) were significantly reduced at T8 compared with the abundance at T0. Furthermore, in the nonsmoking group, 20 genera (28 OTUs) were significantly changed after eight weeks. Among 20 taxa, 14 taxa were increased while the remaining six taxa were reduced at T8 compared with their abundance at T0. The relative abundance of two bacterial taxa, *Turicibacter* and *Streptococcus,* was significantly reduced, whereas that of *Coprococcus* 2 and *Bifidobacterium* was increased after eight weeks regardless of smoking history of navy trainees (Figure 3). Overall, two phyla, namely Firmicutes and Bacteroidetes, were the most modulated phyla after quitting smoking in short periods.

## 4. Discussion

This was an intervention study to determine the impact of changes in healthy lifestyles on gut microbiota modulation. The study was performed by recruiting navy trainees in the naval OCS. The trainees were instructed to stop smoking and drinking alcohol during the eight week training period. In addition, the trainees were ordered to obey the sleep schedule and time of physical exercise. Chiuve et al. [47] discussed that an overall healthy lifestyle includes smoking cessation, alcohol abstinence, regular exercise, and optimal body weight. Such healthy lifestyles have been reported to reduce the risk of several diseases such as cardiovascular disease, diabetes, and cancer [48,49,50]. For comparison, we profiled the metagenomics of fecal samples of healthy civilians, who had no significant lifestyle change in eight weeks. Our study showed that navy trainees, who had a significant change in their healthy lifestyles for eight weeks, had slightly higher OTUs (mean = 119.70, IQR = 95.65–135.10) and Shannon index (mean = 4.93, IQR = 4.58–5.29), though these were not significant, compared with those at baseline (T0), OTUs (mean = 116.60, IQR = 98.03–133.60) and Shannon index (mean = 4.88, IQR = 4.72–5.25) (Figure S1c). Similarly, civilians, who had no significant change in their lifestyle for eight weeks, lacked significant difference between T8 and T0 in all diversity indices (Figure S1c). A lack of significant change in the alpha diversity indices after eight weeks in either of the groups might be attributed to the relatively short period, as the adult gut microbiome is stable and resilient [5]. Furthermore, our study revealed that there was a highly significant microbial community structure among individuals. Notably, host genetics could potentially influence microbial community in the intestine [51]. Genetically-driven intrinsic factors play a role in organizing the gut microbiota structure and formation [52]. Small et al. [53] also investigated the importance of genotype–environment interactions to understand the mechanisms and complex molecular interactions between hosts and their resident microbes. Similar studies have demonstrated that the gut microbiota is influenced by both environmental and genetic factors [54,55,56]. This is due to the fact that intestine could potentially be colonized by microorganisms present in the environment [57]. Nevertheless, the current research data is not sufficient to address the influence of host genetic factors on gut microbiota. The lack of clinical and genomic data from the participants are some of the limitations of this study.

At a lower taxonomic level, the abundance of *Bifidobacterium* spp. was significantly increased in navy trainees at T8 compared with that at T0. This genus is an important component of human gut microbial communities and is known to play an important role in human health [58]. The *Bifidobacterium* genus has been reported to reduce hypercholesterolemia, a major risk factor for cardiovascular disease when taken as probiotics [59] and to improve diabetes when administered to diabetic rats [60]. Furthermore, *Bifidobacterium* spp. regulate intestinal homeostasis, modulate local and systemic immune responses, and protect against inflammatory and infectious diseases [61,62]. *Bifidobacterium* spp. are also associated with a healthier status in adults, and a decrease in *Bifidobacterium* spp. in patients has been associated with severe depression in comparison with people without severe depression [63]. In a different study, the abundance of *Bifidobacterium* was found to be significantly increased in rats who underwent moderate exercise [64]. In contrast, several taxa were significantly decreased in abundance after eight weeks of significant changes in lifestyles. The most reduced genera were *Holdemanella* and *Turicibacter*. Interestingly, these genera were not significantly reduced in the civilian group, who had no significant changes in their lifestyles. *Holdemanella* spp. has been reported to be positively associated with chronic kidney disease [65] and the android fat ratio in male [66], and *Turicibacter* spp. were positively associated with inflammation [67]. Several OTUs belonging to the *Ruminococcus* two genus were significantly reduced while others were enriched after eight weeks. *Ruminococcus* is an enterotype bacteria that enters the intestine [68], has the ability to ferment complex carbohydrates, such as cellulose, pectin, and starch [69,70], and also produces acetate and propionate [71,72]. The *Ruminococcus* genus is heterogeneous and includes both beneficial and harmful species. For example, *Ruminococcus bromii* is known to exert beneficial effects on health [73], whereas other *Ruminococcus* species are proinflammatory [74,75]. Recently *Ruminococcus gnavus* and *R. torques* have been reported to be associated with allergic diseases, Crohn’s disease in infants, and autism spectrum disorders [76,77]. An increase in beneficial *Bifidobacterium* and *Ruminococcus* gut microbes is thought to assist in restoring a heterogeneous balance within the gut and thereby help in the recovery from various diseases or help prevent their occurrence.

Our study also revealed that a significant change in healthy lifestyle factors including physical exercise, loss of weight, and avoiding alcohol in a short period of time (eight weeks) failed to have a significant impact in modulating the gut microbial diversity. However, ceasing smoking showed a substantial impact on the number of observed OTUs. Recently, other studies have also reported the effect of smoking on gut microflora [27,78,79]. The composition of microbial communities is known to differ between the intestines of smokers and non-smokers with a lower diversity present in smokers compared with that in non-smokers [27,78]. Similarly, Bierderman et al. [79] reported that not smoking for eight weeks caused a substantial shift in microbial composition and the increment of microbial diversity. Although several studies have reported the effects of smoking on the gut, there is limited information about the mechanism of the effects of smoking on intestinal microflora [80,81]. Nicotine has been reported to affect mucosal eicosanoids and adherent surface mucus secretion [81], and these changes in the intestinal physical feature may affect the intestinal microbial community [82]. Allais et al. [80] reported that cigarette smoke affects the immune system and thereby causes a shift in the microbiota of the gut. Furthermore, smoking has been reported to enhance oxidative stress and acid–base balance in the gut [83], which in turn influences the intestinal microbiota composition [84].

Here, the navy trainees who had previously smoked but quit for eight weeks were found to have several more abundant genera compared to baseline. OTUs belonging to the *Ruminococcus* 1, *Porphyromonas*, *Mogibacterium*, *Peptoniphilus*, *Murdochiella*, and *Ezakiella* genera were enriched. However, the relative abundance of harmful microorganisms such as *Ruminiclostridium* five and *Clostridium sensu stricto* genera and *Turicibacter* spp. and *Streptococcus* spp. were diminished. *Ruminiclostridium* has been proposed as a reclassification of several *Clostridium* spp. to solve taxonomic problems [85]. *Ruminiclostridium* spp. have been reported to be more abundant in throat cancer patients than healthy subjects [86] and were also more abundant in adults with kidney stones [87]. *Clostridium sensu stricto* one spp. have been reported to cause several human diseases such as tetanus and botulism [88,89]. In addition, *Streptococcus* spp. were found to be enriched in patients with several diseases [90,91]. The *Ruminococcus* one group, which contains 67% of all sequences of *Ruminococcus*, is mainly composed of *R. flavefaciens* and *R. albus* species [92]. Similar to our study, Wang et al. [93] reported that the decrease in the relative abundance of *R. albus* was attributed to smoking. In our study, *Porphyromonas*, which had previously been classified in the genus *Bacteroides* with *Prevotella,* was be more abundant in the navy trainees who quit smoking for eight weeks compared to the abundance at T0 [94]; *Porphyromonas* is part of the salivary microbiome and is found in healthy people [95]. Our results also showed that the *Murdochiella*, *Peptoniphilus*, and *Ezakiella* genera, which belong to the Peptoniphilaceae family, were found to be more abundant after subjects stopped smoking for eight weeks compared with abundance at baseline. This family is known as the human commensal flora [96]. The *Murdochiella* genus was also found to be abundant in healthy children compared with abundance in HIV-infected children [97], and both *Murdochiella* and *Peptoniphilus* genera were also known to be present in distal mucosa among other mucosa and lumen sites in healthy people [98].

## 5. Conclusions

In conclusion, we demonstrated that healthy lifestyles, and particularly quitting smoking, even for short periods, could have a potential positive impact in enhancing the abundance of beneficial microbial taxa and reducing the abundance of harmful microorganisms. Among the various lifestyle changes, stopping smoking for eight weeks resulted in a significant increase in alpha diversity, although a significant change in the other lifestyle factors for eight weeks did not alter the gut microbial diversity. Hence, further intervention studies are warranted to investigate the impacts of combined lifestyle changes for an extended periods on gut microbiota composition. In addition, it is relevant to investigate the long-term effect of healthy lifestyle changes on intestinal bacteria.

## Figures and Tables

**Table 1:** Modifiable-lifestyle factors of civilian and navy trainee groups.

Modifiable Lifestyle Factors	Civilians (*n* = 38)	Navy Trainees (*n* = 66)
Cigarette smoking ^1^ *n* (%)		
Non-smokers (Never smoked)	27 (71.05)	47 (71.21)
Smokers (Currently smokes less than 1 pack per day)	11 (28.95)	14 (21.21)
Alcohol consumption ^1^ *n* (%)		
Nondrinkers (Drinking frequency is less than weekly)	18 (47.37)	28 (42.42)
Regular drinkers (Drinks at least once per week)	18 (47.37)	23 (34.85)
Sleep duration ^1^ *n* (%)		
7–9 h sleep (Recommended hour)	19 (50.00)	52 (78.79)
<7 h sleep (Below recommended hour)	19 (50.00)	9 (13.64)
Physical exercise ^1^, *n* (%)		
Regular exercisers (≥2 h per week)	10 (26.32)	26 (39.39)
Non exercisers (<1 h per week)	28 (73.68)	35 (53.03)
Body mass index (BMI), *n* (%)		
<18.50	2 (5.26)	1 (1.52)
18.50–24.99	26 (68.42)	38 (57.58)
25.00–29.99	10 (26.32)	22 (33.33)

^1^ The percentages do not sum up to 100% because of missing values: five navy trainees for cigarette smoking, sleep time, physical exercise, and body mass index; two civilians and 15 navy trainees for alcohol consumption. *n* refers to the number of subjects participated in each group.

**Table 2:** Characteristics of participants at T0 and T8. Data are shown as mean  ±  SD; *p*-values are obtained by Wilcoxon matched-pairs signed-rank test.

Variables	Civilians (*n* = 38)			Navy Trainees (*n* = 66)		
	T0	T8	*p*-Value	T0	T8	*p*-Value
Cigarette smoking (cigarettes smoked per day)	4.50 ± 7.88	3.90 ± 7.15	0.250	2.28 ± 4.73	0.00 ± 0.00	<0.001
Alcohol consumption (alcohol consumption days per week)	0.83 ± 1.08	0.83 ± 1.08	>0.999	0.90 ± 1.33	0.00 ± 0.00	<0.001
Sleeping duration (hours per day)	6.37 ± 1.30	6.42 ± 1.18	0.856	7.69 ± 1.29	7.57 ± 0.49	0.400
Physical exercise (exercise duration in the past week, min)	84.34 ± 171.20	85.26 ± 155.00	0.587	178.60 ± 228.20	1347.00 ± 203.80	<0.001
Body mass index (BMI, kg/m^2^)	22.91 ± 2.97	22.83 ± 2.92	0.632	24.52 ± 2.59	23.82 ± 2.24	<0.001

Where *n* refers to the number of subjects participated in each group.

**Table 3:** Permutational multivariate analysis of variance (PERMANOVA) table showing the significant effect of individuals in comparison with lifestyle factors on gut microbiota.

Groups	PERMANOVA	
	Pseudo-F	*p*-Value
All participants	4.281	0.001
All participants at T0 vs. T8	0.789	0.771
Civilians	5.120	0.001
Civilians at T0 vs. T8	0.250	1.000
Navy trainees	3.674	0.001
Navy trainees at T0 vs. T8	1.206	0.189

## Data Availability

The datasets generated during and/or analyzed during the current study are available from the NCBI Sequence Read Archive database under accession numbers PRJNA644464.

## Supplementary Materials

The following are available online at https://www.mdpi.com/2076-2607/8/9/1265/s1, Figure S1: Microbial community composition and diversity of all participants at T0 and T8.; Figure S2: Microbial diversity of civilian and navy trainee groups at T0 and T8; Table S1: Baseline characteristics of participants in the study; Table S2: Feature selection analysis for gut microbiome in civilian and navy trainee group at T0 vs T8; Table S3: PERMANOVA showing the effect of subgroups at different time, T0 and T8 in civilian and navy trainee groups; Table S4: Feature selection analysis for gut microbiome in navy smokers and non-smokers at T0 and T8.
