# Supplementary material for: Modulation of Gut Microbiota in Korean Navy Trainees following a Healthy Lifestyle Change
Source: Microorganisms. 2020 Aug 20;8(9):1265. doi: 10.3390/microorganisms8091265 (PMC7569816; doi:10.3390/microorganisms8091265)
Supplement: Supplementary file 1 [file microorganisms-08-01265-s001.pdf]

Supplementary materials

Modulation of gut microbiota in Korean navy trainees following a healthy lifestyle change

YeonGyun Jung [1], Setu Bazie Tagele [1,2], HyunWoo Son [1], Jerald Conrad Ibal [1], Dorsaf Kerfahi [3], Hyunju Yun [4], Bora Lee [4], Clara Yongjoo Park [4], Eun Soo Kim [5], Sang-Jun Kim [6], and Jae-Ho Shin [1,*]

[1] School of Applied Biosciences, Kyungpook National University, Daegu, Republic of Korea; jyg1076@knu.ac.kr (Y.J.); setubazie@gmail.com (S.B.T); thsgusdn2@knu.ac.kr (H.S.); jerald.ibal@gmail.com (J.C.I.)

[2] Department of Applied Plant Sciences, University of Gondar, Ethiopia

[3] Department of Biological Sciences, Keimyung University, Daegu, Republic of Korea; kerfahi.dorsaf@gmail.com

[4] Department of Food and Nutrition, Chonnam National University, Gwangju, Republic of Korea; virt0323@naver.com (H.Y.); lbr1074@naver.com (B.L.); parkcy@jnu.ac.kr (C.Y.P.)

[5] Department of Internal Medicine, School of Medicine, Kyungpook National University, Daegu, Republic of Korea; dandy813@hanmail.net

[6] Department of Natural Sciences, Republic of Korea Naval Academy, Changwon, Republic of Korea; sjkim1963@navy.mil.kr

* Correspondence: jhshin@knu.ac.kr; Tel.: +82-53-950-5716; Fax: +82-53-953-7233

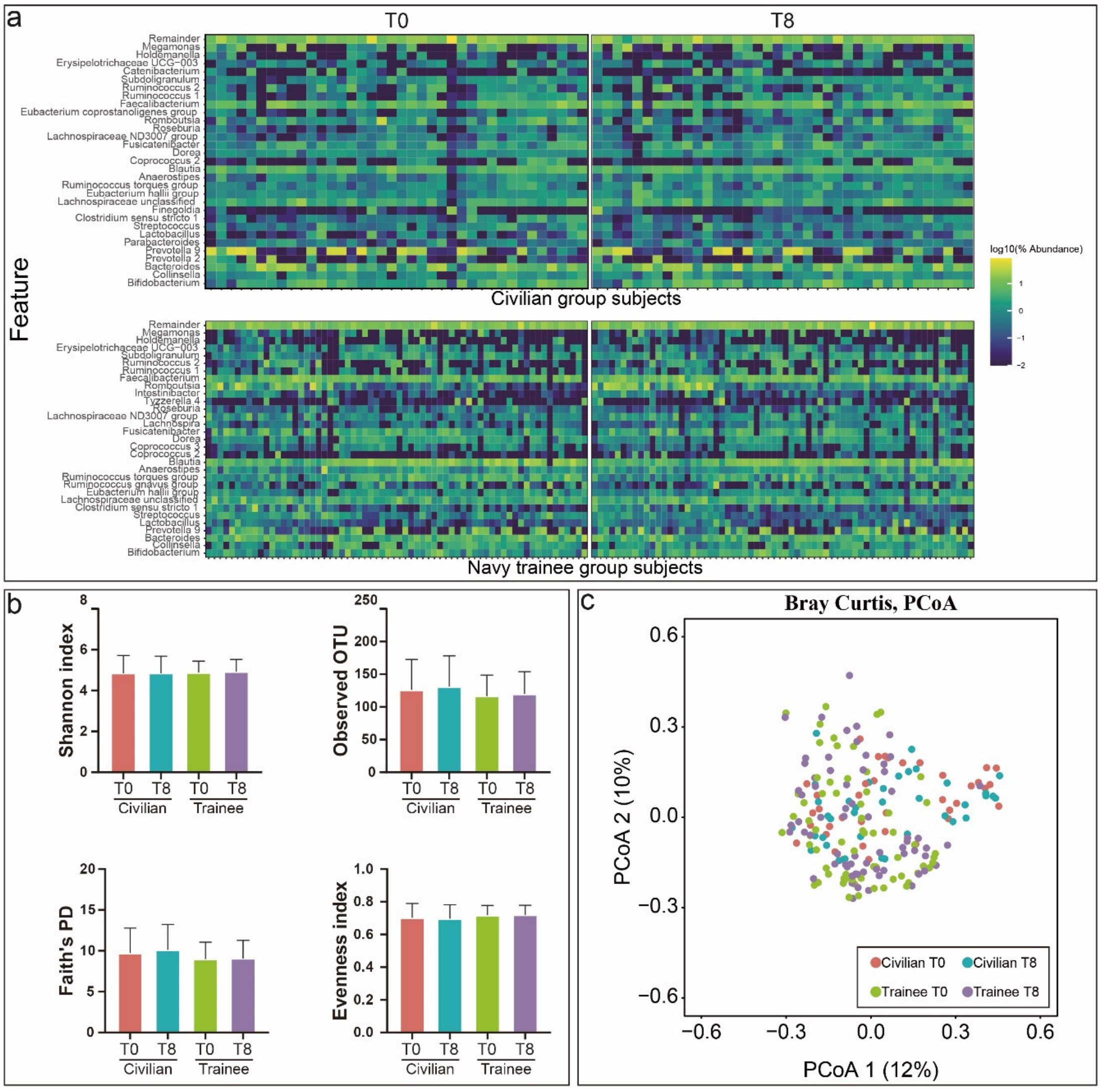

Figure S1. Microbial community composition and diversity of all participants at T0 and T8. (a) A heatmap depicting the abundance of the top 30 genera at T0 and T8 weeks. The color scale represents the log10+1 transformed relative abundance of the number of sequences. (b) Principal component analysis (PCoA) plot based on the Bray–Curtis distance showing the microbiota of civilian and navy trainee groups. (c) α-diversity of microbiota in civilian and navy trainee groups. Data are shown as mean ± SD.

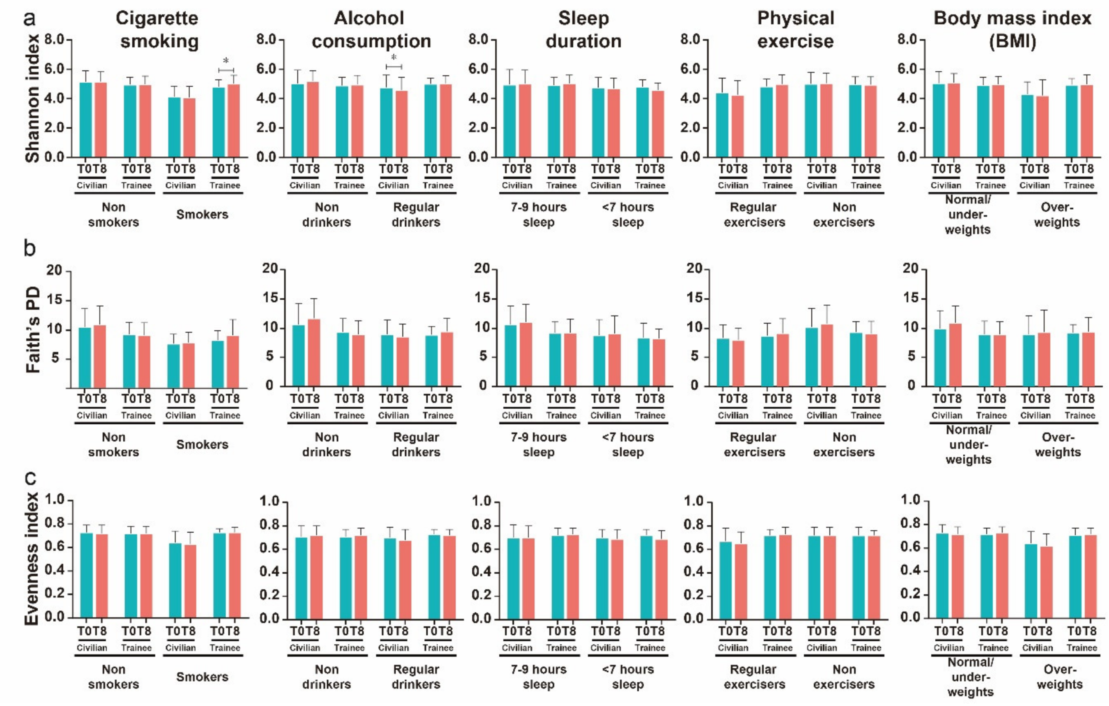

Figure S2. Microbial diversity of civilian and navy trainee groups at T0 and T8. Shannon index (a), Faith's PD (b), and Evenness index (c) in different categories of lifestyle factors in civilian and navy trainee groups at T0 and T8. Values are expressed as means ± SEM. Asterisk (*) indicates statistically significant ($p < 0.05$) differences between groups based on Wilcoxon matched-pairs signed-rank test. At T8, the navy trainees stopped smoking and drinking alcohol, instructed to do physical exercise for 5 h per day and obey an 8 h sleep schedule.

Table S1. Baseline characteristics of participants in the study

Characteristic	Civilian Group (*n* = 38)	Navy trainee Group (*n* = 66)
Gender		
Male *n* (%)	22 (57.89)	55 (83.33)
Female *n* (%)	16 (42.11)	11 (16.67)
Age	30.03 ± 8.76	23.88 ± 1.91
Height, (cm)	169.50 ± 8.41	171.80 ± 6.94
Weight, (kg)	66.42 ± 13.09	72.77 ± 10.94
Body mass index (BMI, kg/m^2)		
< 18.50 *n* (%)	2 (5.26)	1 (1.52)
18.50–24.99 *n* (%)	26 (68.42)	38 (57.58)
25.00–29.99 *n* (%)	10 (26.32)	22 (33.33)
Cigarettes consumption		
Smokers *n* (%)	11 (28.95)	14 (21.21)
Non-smokers *n* (%)	27 (71.05)	47 (71.21)
No Answer *n* (%)	0 (0.00)	5 (7.58)
Cigarettes smoked per day	15.55 ± 6.39	9.93 ± 4.62
Alcohol consumption		
Drinker *n* (%)	18 (47.37)	23 (34.85)
Non-drinker *n* (%)	18 (47.37)	28 (42.42)
No Answer *n* (%)	0 (0.00)	5 (7.58)

Alcohol consumption days per week	1.67 ± 0.97	2.00 ± 1.31
Sleep time (hours per day)	6.51 ± 0.96	7.69 ± 1.29
Sleep quality		
Very Bad *n* (%)	3 (7.89)	0 (0.00)
Bad *n* (%)	3 (7.89)	4 (6.06)
Normal *n* (%)	12 (31.58)	35 (53.03)
Good *n* (%)	16 (42.11)	17 (25.73)
Very Good *n* (%)	4 (10.53)	4 (6.06)
No Answer *n* (%)	0 (0.00)	6 (9.09)
Physical exercise		
Regular exercise for more than 2 hours a week *n* (%)	10 (26.32)	26 (39.39)
Non exercise *n* (%)	28 (73.68)	35 (53.03)
No Answer *n* (%)	0 (0.00)	5 (7.58)
Kind of physical exercise		
Weight training *n* (%)	1 (10.00)	4 (15.38)
Cardio *n* (%)	1 (10.00)	1 (3.85)
Both *n* (%)	8 (80.00)	21 (80.77)
Exercise duration in the past week, (min)	305.5 ± 213.5	390.6 ± 204.2
Number of people living together (*n*)	2.66 ± 1.48	3.38 ± 1.36
Presence of companion animals		
Yes *n* (%)	5 (13.16)	14 (21.21)
No *n* (%)	33 (86.84)	46 (69.70)
No Answer *n* (%)	0 (0.00)	6 (9.09)
Number of bowel movement a week	6.26 ± 3.24	6.75 ± 3.09
Feeling after a bowel movement		
Bad	2 (5.26)	1 (1.52)
Normal	13 (34.21)	28 (42.42)
Good	19 (50.00)	26 (39.39)
Very Good	4 (10.53)	6 (9.09)
No Answer	0 (0.00)	5 (7.58)
Number of days feeling happy in the last week	2.90 ± 2.30	4.32 ± 1.92
Number of days feeling severely depressed past week	1.05 ± 1.51	0.46 ± 1.07
Physical and mental health condition		
Very Bad	1 (2.63)	0 (0)
Bad	10 (26.32)	2 (3.03)
Normal	11 (28.95)	18 (27.27)
Good	14 (36.84)	33 (50)
Very Good	2 (5.26)	6 (9.09)
No Answer	0 (0)	7 (10.61)
Daily intake[1]		
Energy (kcal)	1939.00 ± 749.70	2375.00 ± 901.40
Carbohydrate (g)	286.40 ± 111.40	343.30 ± 141.40
Protein (g)	67.94 ± 30.62	86.64 ± 41.35
Fat (g)	50.27 ± 26.66	61.14 ± 29.46
Cholesterol (g)	313.80 ± 216.00	371.60 ± 199.80
Fiber (mg)	15.72 ± 7.55	19.63 ± 12.93

[1]Daily nutrient intake were adjusted subject number; civilian group (n = 34), navy trainee group (n = 25); FFQ non-responders were excluded. Data are shown as mean ± SD (standard deviation). *n* refers to the number of subjects participated in each group.

Table S2. Feature selection analysis for gut microbiome in civilian and navy trainee group at T0 vs. T8

Group	Phylum	Class	Order	Family	Genus	[1]logFC	[1]logCPM	p-Value	[2]FDR
Civilian group T0 vs. T8	Firmicutes	Bacilli	Lactobacillales	Lactobacillaceae	*Lactobacillus*	−4.608	10.185	2.77E-12	3.22E-10
	Firmicutes	Bacilli	Lactobacillales	Streptococcaceae	*Lactococcus*	4.237	10.070	5.56E-12	3.22E-10
	Actinobacteria	Actinobacteria	Bifidobacteriales	Bifidobacteriaceae	*Gardnerella*	−4.398	10.033	7.55E-12	3.22E-10
	Proteobacteria	Gammaproteobacteria	Pseudomonadales	Pseudomonadaceae	*Pseudomonas*	−4.173	9.895	1.06E-11	3.40E-10
	Firmicutes	Clostridia	Clostridiales	Family XI	*Anaerococcus*	−4.797	11.793	8.85E-10	2.27E-08
	Actinobacteria	Actinobacteria	Propionibacteriales	Propionibacteriaceae	*Cutibacterium*	−3.137	9.604	3.36E-08	5.75E-07
	Firmicutes	Clostridia	Clostridiales	Family XI	*Ezakiella*	−3.941	11.202	3.84E-08	5.75E-07
	Firmicutes	Clostridia	Clostridiales	Family XI	*Anaerococcus*	−3.684	10.302	4.04E-08	5.75E-07
	Firmicutes	Clostridia	Clostridiales	Family XI	*Anaerococcus*	−3.646	10.526	5.10E-08	6.17E-07
	Bacteroidetes	Bacteroidia	Bacteroidales	Porphyromonadaceae	*Porphyromonas*	−3.929	11.017	5.30E-08	6.17E-07
	Firmicutes	Bacilli	Lactobacillales	Streptococcaceae	*Streptococcus*	−3.040	9.384	1.63E-07	1.74E-06
	Firmicutes	Clostridia	Clostridiales	Family XI	*Ezakiella*	−3.947	12.319	4.98E-07	4.91E-06
	Firmicutes	Clostridia	Clostridiales	Family XI	*Peptoniphilus*	−2.398	10.989	6.09E-04	5.01E-03
	Firmicutes	Clostridia	Clostridiales	Family XI	*Peptoniphilus*	−2.184	10.350	6.26E-04	5.01E-03
	Firmicutes	Clostridia	Clostridiales	Christensenellaceae	Christensenellaceae R-7 group	2.000	10.959	1.28E-03	9.62E-03
	Firmicutes	Clostridia	Clostridiales	Family XI	*Peptoniphilus*	−2.343	11.662	1.67E-03	1.17E-02
	Firmicutes	Clostridia	Clostridiales	Lachnospiraceae	*Ruminococcus torques* group	1.923	10.630	1.74E-03	1.17E-02
	Firmicutes	Clostridia	Clostridiales	Lachnospiraceae	*Anaerostipes*	1.712	9.325	2.12E-03	1.36E-02
	Firmicutes	Clostridia	Clostridiales	Lachnospiraceae	*Roseburia*	1.881	12.087	6.55E-03	3.98E-02
	Firmicutes	Clostridia	Clostridiales	Family XI	*Anaerococcus*	−1.874	10.877	6.84E-03	3.98E-02
Navy trainee group T0 vs. T8	Actinobacteria	Actinobacteria	Bifidobacteriales	Bifidobacteriaceae	*Bifidobacterium*	5.184	10.937	7.38E-27	1.05E-24
	Firmicutes	Clostridia	Clostridiales	Family XI	*Murdochiella*	3.494	9.641	1.28E-14	6.05E-13
	Firmicutes	Clostridia	Clostridiales	Family XI	*Peptoniphilus*	3.262	9.352	7.09E-14	2.52E-12
	Firmicutes	Clostridia	Clostridiales	Family XIII	*Mogibacterium*	3.134	9.229	1.92E-13	5.45E-12
	Firmicutes	Erysipelotrichia	Erysipelotrichales	Erysipelotrichaceae	*Turicibacter*	−3.408	10.416	7.91E-13	1.87E-11
	Firmicutes	Clostridia	Clostridiales	Ruminococcaceae	*Ruminococcus* 2	−4.008	11.093	1.02E-12	1.88E-11
	Firmicutes	Clostridia	Clostridiales	Family XIII	S5-A14a	3.055	9.283	1.06E-12	1.88E-11
	Firmicutes	Clostridia	Clostridiales	Lachnospiraceae	*Coprococcus* 2	3.056	9.410	2.32E-12	3.62E-11
	Firmicutes	Clostridia	Clostridiales	Ruminococcaceae	*Ruminococcus* 2	3.591	10.382	2.55E-12	3.62E-11
	Firmicutes	Erysipelotrichia	Erysipelotrichales	Erysipelotrichaceae	*Holdemanella*	−3.622	10.731	1.76E-11	2.27E-10

Firmicutes	Clostridia	Clostridiales	Family XI	*Ezakiella*	2.880	9.863	7.01E-10	8.29E-09
Firmicutes	Bacilli	Lactobacillales	Streptococcaceae	*Streptococcus*	−3.547	11.800	1.46E-09	1.60E-08
Firmicutes	Bacilli	Lactobacillales	Lactobacillaceae	*Lactobacillus*	2.366	12.211	3.08E-09	3.13E-08
Bacteroidetes	Bacteroidia	Bacteroidales	Bacteroidaceae	*Bacteroides*	2.516	9.536	8.94E-09	8.47E-08
Firmicutes	Bacilli	Lactobacillales	Streptococcaceae	*Streptococcus*	−2.129	13.592	2.80E-08	2.34E-07
Firmicutes	Clostridia	Clostridiales	Ruminococcaceae	CAG-352	−2.289	9.357	1.83E-07	1.44E-06
Firmicutes	Bacilli	Lactobacillales	Streptococcaceae	*Lactococcus*	−2.619	11.278	4.11E-07	3.06E-06
Firmicutes	Clostridia	Clostridiales	Family XI	*Peptoniphilus*	2.270	9.706	4.31E-07	3.06E-06
Firmicutes	Bacilli	Lactobacillales	Lactobacillaceae	*Lactobacillus*	3.106	12.635	8.78E-07	5.94E-06
Firmicutes	Clostridia	Clostridiales	Peptostreptococcaceae	*Romboutsia*	-2.231	9.890	1.28E-06	8.29E-06
Firmicutes	Clostridia	Clostridiales	Family XI	*Ezakiella*	2.657	11.157	1.40E-06	8.66E-06
Firmicutes	Clostridia	Clostridiales	Lachnospiraceae	*Coprococcus* 2	2.237	10.821	5.96E-06	3.53E-05
Firmicutes	Clostridia	Clostridiales	Family XI	*Anaerococcus*	2.139	10.496	1.43E-05	8.11E-05
Firmicutes	Erysipelotrichia	Erysipelotrichales	Erysipelotrichaceae	*Catenibacterium*	1.587	9.311	3.32E-04	1.75E-03
Proteobacteria	Gammaproteobacteria	Enterobacteriales	Enterobacteriaceae	*Escherichia-Shigella*	−1.324	11.522	3.34E-04	1.75E-03
Bacteroidetes	Bacteroidia	Bacteroidales	Prevotellaceae	*Prevotella 9*	1.595	9.679	6.57E-04	3.33E-03
Bacteroidetes	Bacteroidia	Bacteroidales	Bacteroidaceae	*Bacteroides*	−1.824	13.441	7.35E-04	3.60E-03
Firmicutes	Clostridia	Clostridiales	Lachnospiraceae	*Blautia*	−1.279	10.532	9.19E-04	4.35E-03
Firmicutes	Clostridia	Clostridiales	Lachnospiraceae	*Roseburia*	1.719	12.225	1.48E-03	6.77E-03
Firmicutes	Bacilli	Lactobacillales	Lactobacillaceae	*Lactobacillus*	−1.462	10.450	1.62E-03	7.17E-03
Bacteroidetes	Bacteroidia	Bacteroidales	Tannerellaceae	*Parabacteroides*	−1.379	10.738	1.71E-03	7.37E-03
Firmicutes	Erysipelotrichia	Erysipelotrichales	Erysipelotrichaceae	*Coprobacillus*	−1.355	9.598	1.90E-03	7.94E-03
Firmicutes	Clostridia	Clostridiales	Ruminococcaceae	*Ruminococcaceae* UCG-013	0.931	12.157	3.17E-03	1.29E-02
Bacteroidetes	Bacteroidia	Bacteroidales	Prevotellaceae	*Alloprevotella*	−1.383	10.235	3.73E-03	1.47E-02
Firmicutes	Bacilli	Lactobacillales	Leuconostocaceae	*Weissella*	1.316	10.507	4.14E-03	1.59E-02
Firmicutes	Clostridia	Clostridiales	Ruminococcaceae	*Eubacterium coprostanoligenes* group	1.325	10.815	6.39E-03	2.33E-02
Firmicutes	Clostridia	Clostridiales	Family XI	*Anaerococcus*	1.495	11.156	6.67E-03	2.36E-02
Firmicutes	Clostridia	Clostridiales	Ruminococcaceae	*Faecalibacterium*	−1.263	9.643	6.82E-03	2.36E-02
Firmicutes	Clostridia	Clostridiales	Clostridiaceae 1	*Clostridium sensu stricto* 1	1.039	10.881	1.38E-02	4.68E-02

[1]logFC and logCPM refers to log fold change and log value of computes counts per million, respectively.

[2]FDR refers to false discovery rate and was computed using edgeR package in R.

Table S3. Permutational multivariate analysis of variance (PERMANOVA) showing the effect of subgroups at different time, T0 and T8 in civilian and navy trainee groups.

Subgroups	PERMANOVA					
	Civilian group			Navy trainee group		
	pseudo-F	p-value	q-value	pseudo-F	p-value	q-value
Non-Smokers T0 vs. T8	0.392	1.000	1.000	0.899	0.596	0.650
Smokers T0 vs. T8	0.131	1.000	1.000	0.629	0.922	0.976
Non-drinkers T0 vs. T8	0.311	1.000	1.000	0.613	0.947	1.000
Regular-drinkers T0 vs. T8	0.287	1.000	1.000	0.920	0.577	0.689
7–9 hours sleep T0 vs. T8	0.467	0.995	0.995	1.118	0.288	0.384
< 7 hours sleep T0 vs. T8	0.425	0.991	0.995	1.312	0.386	0.463
Regular-exercisers T0 vs. T8	0.245	0.997	1.000	0.786	0.769	0.814
Non-exerciser T0 vs. T8	0.238	1.000	1.000	0.954	0.496	0.541
Normal/underweight T0 vs. T8	0.497	0.989	1.000	0.533	0.965	1.000
Overweight T0 vs. T8	0.294	0.995	1.000	1.089	0.314	0.480

9 **Table S4.** Feature selection analysis for gut microbiome in navy smokers and non-smokers at T0 vs. T8

Subgroup	Phylum	Class	Order	Family	Genus	[1]logFC	[1]logCPM	p-value	[2]FDR
Smokers	Firmicutes	Clostridia	Clostridiales	Family XI	*Ezakiella*	6.94	12.34	6.15E-07	6.52E-05
	Firmicutes	Clostridia	Clostridiales	Family XI	*Ezakiella*	5.73	11.19	2.22E-06	8.22E-05
	Actinobacteria	Actinobacteria	Bifidobacteriales	Bifidobacteriaceae	*Bifidobacterium*	5.82	11.27	2.33E-06	8.22E-05
	Firmicutes	Clostridia	Clostridiales	Family XI	*Murdochiella*	5.70	11.30	4.38E-06	9.89E-05
	Firmicutes	Clostridia	Clostridiales	Family XIII	S5-A14a	5.37	10.85	5.41E-06	9.89E-05
	Firmicutes	Clostridia	Clostridiales	Family XIII	*Mogibacterium*	5.39	10.87	5.67E-06	9.89E-05
	Firmicutes	Clostridia	Clostridiales	Family XI	*Peptoniphilus*	5.45	10.92	6.53E-06	9.89E-05
	Firmicutes	Clostridia	Clostridiales	Lachnospiraceae	*Coprococcus 2*	5.45	10.93	8.73E-06	1.16E-04
	Firmicutes	Erysipelotrichia	Erysipelotrichales	Erysipelotrichaceae	*Turicibacter*	-4.11	10.67	2.49E-05	2.79E-04
	Firmicutes	Clostridia	Clostridiales	Ruminococcaceae	*Ruminiclostridium* 5	-4.45	10.25	4.22E-05	4.06E-04
	Bacteroidetes	Bacteroidia	Bacteroidales	Porphyromonadaceae	*Porphyromonas*	4.44	10.03	4.63E-05	4.09E-04
	Firmicutes	Clostridia	Clostridiales	Ruminococcaceae	*Ruminococcus* 1	4.03	9.95	1.12E-04	9.12E-04
	Firmicutes	Bacilli	Lactobacillales	Streptococcaceae	*Streptococcus*	-5.32	13.80	2.91E-04	2.20E-03
	Firmicutes	Clostridia	Clostridiales	Family XI	*Peptoniphilus*	3.98	11.19	6.00E-04	4.24E-03
	Firmicutes	Bacilli	Lactobacillales	Streptococcaceae	*Streptococcus*	-2.89	12.59	1.34E-03	8.87E-03
	Firmicutes	Clostridia	Clostridiales	Clostridiaceae 1	*Clostridium sensu stricto* 1	-3.88	14.05	1.72E-03	1.07E-02
Non-smokers	Actinobacteria	Actinobacteria	Bifidobacteriales	Bifidobacteriaceae	*Bifidobacterium*	5.42	10.93	1.88E-22	2.55E-20
	Firmicutes	Erysipelotrichia	Erysipelotrichales	Erysipelotrichaceae	*Holdemanella*	-5.45	10.89	8.97E-17	6.10E-15
	Firmicutes	Bacilli	Lactobacillales	Streptococcaceae	*Lactococcus*	3.47	9.98	1.04E-12	3.55E-11
	Bacteroidetes	Bacteroidia	Bacteroidales	Prevotellaceae	*Prevotella* 9	-3.16	9.15	4.10E-10	1.04E-08
	Firmicutes	Erysipelotrichia	Erysipelotrichales	Erysipelotrichaceae	*Turicibacter*	-3.70	10.84	4.58E-10	1.04E-08
	Bacteroidetes	Bacteroidia	Bacteroidales	Prevotellaceae	*Prevotella* 9	3.11	9.19	1.02E-09	1.99E-08
	Firmicutes	Clostridia	Clostridiales	Ruminococcaceae	*Ruminococcus* 2	-4.23	11.60	1.61E-09	2.74E-08
	Firmicutes	Clostridia	Clostridiales	Ruminococcaceae	*Ruminococcus* 2	3.72	10.82	8.51E-09	1.29E-07
	Bacteroidetes	Bacteroidia	Bacteroidales	Bacteroidaceae	*Bacteroides*	2.88	9.79	1.12E-07	1.52E-06
	Bacteroidetes	Bacteroidia	Bacteroidales	Prevotellaceae	*Prevotella* 9	2.72	9.41	2.21E-07	2.73E-06
	Firmicutes	Clostridia	Clostridiales	Ruminococcaceae	CAG-352	-2.47	9.69	7.23E-06	7.56E-05
	Firmicutes	Bacilli	Lactobacillales	Streptococcaceae	*Lactococcus*	-2.60	11.66	3.87E-05	3.76E-04
	Firmicutes	Bacilli	Lactobacillales	Lactobacillaceae	*Lactobacillus*	1.54	11.42	5.34E-05	4.84E-04
	Firmicutes	Bacilli	Lactobacillales	Lactobacillaceae	*Lactobacillus*	3.02	13.22	8.82E-05	7.50E-04
	Firmicutes	Bacilli	Lactobacillales	Streptococcaceae	*Streptococcus*	-1.73	13.57	1.26E-04	1.01E-03

Firmicutes	Clostridia	Clostridiales	Ruminococcaceae	*Eubacterium coprostanoligenes* group	1.99	9.83	2.35E-04	1.77E-03
Proteobacteria	Gammaproteobacteria	Enterobacteriales	Enterobacteriaceae	*Escherichia-Shigella*	-1.57	11.65	3.77E-04	2.70E-03
Firmicutes	Clostridia	Clostridiales	Peptostreptococcaceae	*Romboutsia*	-1.83	9.94	1.08E-03	7.31E-03
Firmicutes	Erysipelotrichia	Erysipelotrichales	Erysipelotrichaceae	*Coprobacillus*	-1.72	10.01	1.91E-03	1.14E-02
Firmicutes	Bacilli	Lactobacillales	Lactobacillaceae	*Lactobacillus*	-1.78	10.73	1.93E-03	1.14E-02
Bacteroidetes	Bacteroidia	Bacteroidales	Prevotellaceae	*Alloprevotella*	-1.77	10.65	2.90E-03	1.64E-02
Firmicutes	Erysipelotrichia	Erysipelotrichales	Erysipelotrichaceae	*Catenibacterium*	1.64	9.65	3.02E-03	1.64E-02
Firmicutes	Bacilli	Lactobacillales	Leuconostocaceae	*Weissella*	1.57	10.51	3.29E-03	1.72E-02
Firmicutes	Clostridia	Clostridiales	Ruminococcaceae	*Ruminococcaceae* UCG-013	1.13	12.26	3.98E-03	2.01E-02
Firmicutes	Clostridia	Clostridiales	Lachnospiraceae	*Coprococcus* 2	1.62	10.76	5.26E-03	2.55E-02
Bacteroidetes	Bacteroidia	Bacteroidales	Prevotellaceae	*Prevotella* 9	1.62	10.06	5.45E-03	2.55E-02
Firmicutes	Clostridia	Clostridiales	Ruminococcaceae	UBA1819	-1.44	10.94	9.37E-03	4.25E-02
Bacteroidetes	Bacteroidia	Bacteroidales	Bacteroidaceae	*Bacteroides*	-1.67	13.59	9.69E-03	4.25E-02

[1]logFC and logCPM refers to log fold change and log value of computes counts per million, respectively.[2]FDR refers to false discovery rate and was computed using edgeR package in R.
